# MAFF Expression Reshapes Key Stress-Response Proteins During Light-Induced Injury

**DOI:** 10.3390/antiox15101354

**Published:** 2026-10-20

**Authors:** Jialing Zhang, Ting Zhang, Azhar Dzulhadj B. Arafah, Sora Lee, Michelle Yam, Jingwen Zeng, Chen-Chin Chang, Xiaosuo Wang, Mark C. Gillies, Ling Zhu

**Affiliations:** 1 Macula Research Group, Save Sight Institute, Faculty of Medicine and Health, The University of Sydney, Sydney 2050, Australia; jzha4608@uni.sydney.edu.au (J.Z.);; 2 Cardiometabolic Medicine Group, School of Medical Sciences, Faculty of Medicine and Health, Charles Perkins Centre, The University of Sydney, Sydney 2050, Australia

**Keywords:** MAFF, Müller cell, Antioxidants, light stress, oxidative stress, retina, macular diseases, proteomic

## Abstract

MAFF is a small MAF family transcription factor involved in antioxidant signaling and cytoprotective gene regulation. MAFF is differentially expressed across retinal regions, with particularly low expression in macular Müller cells, which play a central role in retinal redox balance. Reduced MAFF expression may weaken macular resilience to oxidative stress (OS), increasing susceptibility to age-related macular degeneration (AMD) and macular telangiectasia type 2. However, the function of MAFF in Müller cells remains unclear. Human primary Müller cells (huPMCs) from non-diseased human donor eyes were used to investigate MAFF modulation under stress. Cells were transfected with MAFF plasmid DNA to evaluate intracellular reactive oxygen species (ROS) following H_2_O_2_-induced stress. For functional and proteomic analyses, MAFF was overexpressed using mRNA or knocked down using siRNA and validated by Western blot. huPMCs were exposed to light stress and cell viability was assessed using the AlamarBlue assay. Molecular changes were characterized by liquid chromatography–tandem mass spectrometry (LC-MS/MS)-based quantitative proteomics. MAFF upregulation significantly reduced intracellular ROS following H_2_O_2_-induced stress. MAFF protein expression decreased following siRNA-mediated knockdown (KD) and increased following mRNA-mediated overexpression (OE). MAFF OE improved Müller cell viability under light stress, whereas MAFF KD reduced viability. Exploratory proteomic profiling quantified 5907 proteins and identified candidate molecular changes associated with MAFF expression. Pathway analyses suggested that reduced MAFF expression was associated with oxidative-stress response signatures, whereas elevated MAFF expression was associated with changes in mitochondrial and immune/inflammatory pathways. KEAP1, BMP2K, VPS28, and PDCD4 were negatively associated with MAFF expression, suggesting potential regulation of antioxidant defense, vesicle trafficking, and apoptosis-related processes. Elevated MAFF expression was associated with reduced ROS accumulation and improved cell viability, while exploratory proteomic analysis identified changes in mitochondrial, oxidative-stress, and immune/inflammatory pathways. These findings support further investigation of MAFF as a contributor to macular stress susceptibility and as a candidate for future therapeutic studies.

## 1. Introduction

Musculoaponeurotic fibrosarcoma oncogene homolog (MAF) was first identified in 1989 during the analysis of the genome of the AS42 avian transforming retrovirus by Nishizawa et al. It was established that the MAF gene encodes a basic-leucine zipper (BZIP) transcription factor [1]. Small MAF proteins, including MAF BZIP Transcription Factor F (MAFF), MAF BZIP Transcription Factor K (MAFK), and MAF BZIP Transcription Factor G (MAFG), lack the transactivation domains found in larger MAF proteins [2]. These proteins primarily act as transcriptional regulators by forming dimers with other bZip proteins and binding to MAF Recognition Elements (MAREs) in DNA [3,4]. Small MAF proteins interact with Fos Proto-Oncogene (FOS) to form heterodimers or homodimerize themselves. These complexes act as competitive repressors of NF-E2 family members, thereby playing a significant role in regulating transcriptional activity [4]. Later studies revealed that small MAF proteins can also form heterodimers with Cap‘n’Collar (CNC) family members, such as NFE2-Like BZIP Transcription Factor 1 (NRF1) and NFE2-Like BZIP Transcription Factor 2 (NRF2) [5]. These interactions activate antioxidant response genes, protecting cells against oxidative stress (OS) and maintaining redox homeostasis [5].

Small MAF proteins are increasingly recognized as important regulators of disease-related transcriptional programs. In cancer, small MAF proteins cooperate with transcription factors such as BACH Transcriptional Regulator 1 (BACH1) and NRF2 to regulate gene networks involved in tumor progression, metastasis, epithelial–mesenchymal transition, extracellular matrix remodeling and antioxidant defense [6,7,8,9,10]. Consistent with this, elevated MAFF expression has been reported in liver cancer cells, where it promotes tumor metastasis through recruitment of the TIP60 chromatin remodeling complex [11]. In the cardiovascular system, reduced MAFF expression has been associated with more severe coronary artery disease and dysregulation of cholesterol metabolism and inflammatory responses [12]. In the nervous system, deficiencies in MAFG and MAFK have been linked to progressive motor dysfunction, potentially due to impaired glycine receptor expression, an essential inhibitory neurotransmitter receptor in the central nerve system. Small MAF mutations are associated with dysregulation of BACH1 and Heme Oxygenase 1 (HMOX1), leading to OS and disruption of antioxidant signaling pathways, which may contribute to neurodegenerative diseases [13]. In the eye, a mutation in the MAF gene (c.812 T > A, p.Val271Glu) has been implicated in congenital nuclear cataract, disrupting the transactivation of critical lens-related genes, which are essential for maintaining lens clarity and preventing protein aggregation. Additionally, this mutation affects non-crystallin genes such as HMOX1, a key player in antioxidant defense, and KDEL Endoplasmic Reticulum Protein Retention Receptor 2, which regulates endoplasmic reticulum (ER) stress [14].

The retina is the thin layer of tissue lining the internal surface of the back of the eye that detects light and converts it into neural signals that are transmitted to the brain. Among the various cell types in the retina, Müller glial cells are unique in that they span the entire thickness of the retina, providing both structural and functional support to retinal neurons while maintaining overall retinal homeostasis [15]. The transcription factors MAFG and MAFK are classified as having low cell specificity, with broad expression across various retinal cell types. In contrast, MAFF is more cell-specific, with notable enrichment in Müller glial cells in which its expression reaches 48.5 normalized transcripts per million (nTPM) based on single-cell RNA expression data from the Human Protein Atlas [16]. While the specific role of MAFF in ocular and nervous system function has not been clearly defined, MAFK and MAFG have been extensively studied and shown to be associated with cataracts and neurodegenerative diseases.

We have previously reported that MAFF expression differs significantly across retinal regions, with substantially lower expression in the human macula than in the peripheral retina [17]. This is notable because the macula is highly susceptible to OS-related diseases such as age-related macular degeneration (AMD) and macular telangiectasia type 2 [18,19]. Reduced MAFF expression may weaken the ability of macular Müller cells to manage OS, increasing vulnerability to degeneration. Therefore, this study aimed to define the function of MAFF in human primary Müller cells (huPMCs) using gain- and loss-of-function approaches combined with quantitative proteomics. Understanding MAFF function may provide insight into the potential contribution of MAFF to macular susceptibility to OS.

## 2. Materials and Methods

### 2.1. Primary Müller Cell Culture

Human retinas were obtained from ten pairs of post-mortem donor eyes (donor information provided in Table A1) with no history of ocular disease, following ethical approval from the Human Research Ethics Committee of the University of Sydney (HREC#:16/282). huPMCs were isolated from donor eyes and cultured according to a previously established protocol [20]. Briefly, isolated retinas were incubated overnight at 4 °C in DMEM (Gibco, Thermo Fisher Scientific, Waltham, MA, USA), followed by enzymatic digestion with TrypLE (Thermo Fisher, Waltham, MA, USA) at 37 °C for 60 min. Retinal tissue was then dissected into approximately 1 × 1 mm fragments and transferred to T25 culture flasks (Corning, New York, NY, USA) for Müller cell outgrowth. huPMCs were cultured in DMEM supplemented with 10% fetal bovine serum (FBS) and 1% penicillin–streptomycin (Invitrogen, Thermo Fisher Scientific, Waltham, MA, USA). To maintain consistency and minimize variability, all experiments were conducted using cells at passage three (P3). The transition from the initial passage (P0) to P3 required approximately 12 weeks to establish a stable and well-characterized huPMC culture system.

### 2.2. MAFF Plasmid DNA (pDNA)/Messenger RNA(mRNA)/Small Intefering RNA (siRNA) Transfection

huPMCs at P3 and 80% confluency were used for MAFF overexpression (OE) (*n* = 4 donors) and knockdown (KD) (*n* = 3 donors) experiments. For the pilot reactive oxygen species (ROS) assay, cells were transfected with MAFF plasmid DNA (pDNA) or control GFP-tagged pDNA (GenScript, Piscataway, NJ, USA) using Lipofectamine 3000 reagent (#L3000008; Thermo Fisher, Waltham, MA, USA) at a final concentration of 1 µg/mL. For subsequent functional validation and proteomic analysis, MAFF OE was performed using synthesized MAFF mRNA (GenScript, Piscataway, NJ, USA) or an EGFP mRNA control (#L7601, CleanCap, TriLink BioTechnologies, San Diego, CA, USA) with MessengerMAX reagent (#LMRNA003, Invitrogen, Thermo Fisher Scientific, Waltham, MA, USA) at a final concentration of 1 µg/mL. MAFF KD was performed using siRNA treatment in P3 huPMCs as previously described [17], at a final concentration of 40 nM. pDNA-transfected cells were used for intracellular ROS detection. mRNA- and siRNA-treated cells were seeded in 48-well plates (1 × 10^4^ cells/well) for AlamarBlue assays and Western blot (WB) validation, and in 6-well plates (8 × 10^4^ cells/well for subsequent proteomic analysis.

### 2.3. Western Blot (WB)

MAFF protein expression was assessed in huPMCs from one representative donor following mRNA or siRNA transfection using WB analysis. Cells were lysed in radioimmunoprecipitation assay (RIPA) buffer (#900000064244, Sigma-Aldrich, St. Louis, MO, USA) supplemented with phosphatase and protease inhibitors (1:100, #5872S, Cell Signaling Technologies, Danvers, MA, USA). Cell lysates were centrifuged at 12,000× *g* at 4 °C for 15 min, and supernatants were collected. 5 µ/uL proteins were mixed with dithiothreitol (DTT; #D9779-10G, Sigma-Aldrich, St. Louis, MO, USA) and loading buffer (#NP0007, Invitrogen, Thermo Fisher Scientific, Waltham, MA, USA), heated at 75 °C for 10 min, separated by gel electrophoresis, and transferred onto polyvinylidene fluoride (PVDF) membranes (#IEVH85R, Merck Millipore, Burlington, MA, USA) using a wet transfer system (#1703930, Bio-Rad, Hercules, CA, USA). Membranes were blocked with 5% bovine serum albumin (BSA; #A9647-100G, Sigma-Aldrich, St. Louis, MO, USA) in tris-buffered saline (TBS) for 1 h at room temperature and incubated with primary antibodies (Table 1) diluted in 1% bovine serum albumin in tris-buffered saline with Tween 20 (TBST) overnight at 4 °C. Membranes were then incubated with horseradish peroxidase (HRP)-conjugated secondary antibodies (Table 1) diluted in 1% bovine serum albumin in tris-buffered saline with Tween 20 for 2 h at room temperature. Protein bands were visualized using Clarity enhanced chemiluminescence (ECL) substrate (#170-5061, Bio-Rad, Hercules, CA, USA) and imaged using the G-Box imaging system (In Vitro Technologies, Noble Park North, VIC, Australia). Band intensities were quantified using GeneTools software (version 4.3.17) and normalized to α/β tubulin.

### 2.4. Light/Hydrogen Peroxide (H_2_O_2_) Stress After MAFF Modulation Treatments

Following 48 h of pDNA transfection, huPMCs (P3) were treated with hydrogen peroxide (H_2_O_2_) to induce OS. huPMCs were treated with 10 mM H_2_O_2_, with 0 μM H_2_O_2_ used as the unstressed control. For RNA-based MAFF modulation experiments, huPMCs were exposed to light stress after 24 h of mRNA transfection or 72 h of siRNA transfection. One plate was wrapped in foil to serve as the dim-light control (5 lux), while another identically treated plate was exposed to light stress (32,000 lux) for 4 h using a custom-built light exposure system within the tissue culture incubator, as previously described [17].

### 2.5. Intracellular ROS and Cell Viability Assays

huPMCs (P3) cultured in 48-well plates (Corning, New York, NY, USA) were used to detect ROS and AlamarBlue cell viability assays. Intracellular ROS levels were measured using the Reactive Oxygen Species Assay Kit (#ab186027, Abcam, Cambridge, UK), while cell viability was measured using the AlamarBlue (#DAL1025; Thermo Fisher, Waltham, MA, USA), following the manufacturers’ instructions. Fluorescence signals were measured using a CLARIOstar Plus plate reader (BMG Labtech, Ortenberg, Germany) at excitation/emission wavelengths of 520/605 nm for the ROS assay and 544/590-10 nm for the AlamarBlue assay.

### 2.6. Protein Extraction from Treated huPMCs for Proteomic Analysis

Following mRNA or siRNA transfections, huPMCs in the 6-well plate were lysed and proteins were extracted using a SIMPLEX-based protocol [21], but with a modification of methanol:chloroform:water ratio to 2:2:1. huPMCs cultured in 6-well plates were placed on ice, and 200 µL of pre-chilled methanol (−30 °C; #FSBA456-4; Thermo Fisher, Waltham, MA, USA) was added directly to each well. huPMCs were scraped and transferred to pre-chilled 1.7 mL microcentrifuge tubes (Axygen, Corning Incorporated, Corning, New York, NY, USA). Cold chloroform (#650498; Sigma-Aldrich, St. Louis, MO, USA) was added, samples were vortexed for 30 s, followed by addition of 100 µL HPLC-grade water and further vortexing for 30 s. Samples were centrifuged at 15,000 × *g* for 20 min at 4 °C (5415R; Eppendorf, Hamburg, Germany) to obtain a biphasic system with a visible interfacial protein layer. The protein interphase was collected and retained for proteomic analysis.

### 2.7. Protein Sample Preparation Before Loading

The protein pellet from the interphase was precipitated by adding four volumes of ice-cold acetone (#179124; Sigma-Aldrich, St. Louis, MO, USA) and incubating overnight at −30 °C. Samples were then centrifuged at 9000× *g* for 20 min at 4 °C, and the protein pellet was air-dried without over-drying. The dried pellet was resuspended in 6 M urea (#U6504; Sigma-Aldrich, St. Louis, MO, USA)/2 M thiourea (#T7875; Sigma-Aldrich, St. Louis, MO, USA) in 100 mM HEPES buffer (#83264; Sigma-Aldrich, St. Louis, MO, USA), followed by reduction with dithiothreitol (#D9163; Sigma-Aldrich, St. Louis, MO, USA) and alkylation with iodoacetamide (#I1149; Sigma-Aldrich, St. Louis, MO, USA). Protein concentration was measured using the Qubit Protein Assay (#Q33212; Thermo Fisher, Waltham, MA, USA). Equal amounts of protein were diluted in 50 mM ammonium bicarbonate (#09830; Sigma-Aldrich, St. Louis, MO, USA) and digested overnight at 37 °C with sequencing-grade trypsin. The resulting peptides were acidified with trifluoroacetic acid (#T6508; Sigma-Aldrich, St. Louis, MO, USA), purified using C18 ZipTips (#ZTC18S960; Merck Millipore, Burlington, MA, USA), and dried by a freeze dryer (Alpha 2-4 LSCbasic; Scitek, Lane Cove West, NSW, Australia).

### 2.8. Peptide Processing and Liquid Chromatography–Tandem Mass Spectrometry (LC-MS/MS)

Dried peptide samples were reconstituted in loading buffer (3% ACN, 0.1% FA in water; *v*/*v*) followed by sonication and centrifugation at 16,000× *g* for 10 min at 4 °C. Peptides were analyzed using an Ultimate 3000 UHPLC system coupled to a Q Exactive HFX Orbitrap mass spectrometer (Thermo Fisher, Waltham, MA, USA) at the Sydney Mass Spectrometry facility. 1 µg of peptides were injected at a volume of 3 μL from a 96-well skirted plate (#AB-0800-L, Thermo Fisher, Waltham, MA, USA) sealed with an Axymat silicone sealing mat (#AM-96-PCR-RD).

Chromatographic separation was performed using an in-house packed reversed-phase C18 analytical column (PUR 120 C18, 1.9 μm particles, 50 cm in length, #r119.aq). The column was maintained at 60 °C and operated at a flow rate of 300 nL/min. Mobile phase A consisted of 0.1% formic acid in Optima LC/MS-grade water (#W6-4, Thermo Fisher, Waltham, MA, USA), while mobile phase B contained 80% ACN, 19.9% water, and 0.1% formic acid. Peptides were separated using a 90-min LC linear gradient prior to mass spectrometric acquisition.

Mass spectrometric data were acquired in positive ion mode using Xcalibur software (version 4.1.31.9). Full MS scans were collected over an *m*/*z* range of 350–1550 at a resolution of 70,000, followed by data-independent acquisition (DIA) MS/MS scans at a resolution of 17,500 with variable *m*/*z* windows covering the mass range of 300–900. System performance and reproducibility were monitored using 30 fmol predigested BSA peptide standards (#P8108S, GeneSearch, Arundel, QLD, Australia) analyzed at the beginning and end of each batch.

### 2.9. Proteomic Data Analysis

Following data acquisition, raw proteomic files were processed using Spectronaut (version 21, Biognosys, Schlieren, Switzerland) with the directDIA analysis workflow. The raw mass spectrometry data were deposited in the ProteomeXchange Consortium through the iProX partner repository under the dataset identifier PXD080464. The dataset remained under embargo during peer review and will be released publicly upon publication. The resulting protein (listed as gene name) intensity matrix was exported for downstream analysis. Missing intensity values were imputed using a downshifted Gaussian approach [22]. Briefly, protein intensities were first transformed to log_2_ scale to stabilize variance and approximate normal distribution. For each protein, the mean (μ) and standard deviation (σ) of observed values were calculated, and missing entries were replaced with random values drawn from a normal distribution defined as N (μ − 1.8σ, 0.3σ). This approach places imputed values in the lower tail of the intensity distribution, reflecting the assumption that missing values arise from low-abundance signals below the detection threshold. For exploratory pairwise proteomic comparisons, paired two-tailed Student’s *t*-tests were used to assess within-donor differences between predefined experimental conditions, thereby accounting for the donor-matched study design. Proteins meeting the predefined criteria of nominal *p* < 0.05 and |log_2_ fold-change| > 1 were considered exploratory candidate proteins for subsequent pathway and functional analyses. In addition, a factorial limma analysis with empirical Bayes moderation was performed on 5737 proteins retained after duplicated protein entries were consolidated by averaging their log_2_-transformed abundance values. Donor was incorporated into the model to account for the paired donor structure, while MAFF treatment, light exposure, and the MAFF × light interaction were explicitly modelled. Benjamini–Hochberg correction was applied for multiple testing. The results of the limma analysis are provided in Supplementary Table S1.

### 2.10. Partial Least Squares Discriminant Analysis (PLS-DA)

PLS-DA was performed in RStudio using R version 2025.09.2+418 with the mixOmics package. Data were log_2_-transformed and scaled before multivariate analysis. PLS-DA was performed using the plsda() function, with experimental groups defined according to MAFF expression status and light exposure condition. The first two components were visualized using score plots to assess group separation. Exported Variable Importance in Projection (VIP) score for each comparison group were listed in appendix (Table A2). Given the limited number of biologically independent donors, PLS-DA was used as an exploratory visualization rather than as a formal inferential analysis. Of the 5907 quantified proteins, 5737 were retained for the R-based analysis after duplicated protein entries were consolidated by averaging their log_2_-transformed abundance values. Model robustness and potential overfitting were assessed by permutation testing, with validation results provided in Table S2.

### 2.11. QIAGEN Ingenuity Pathway Analysis (IPA)

QIAGEN IPA was performed to identify enriched canonical pathways and predict pathway activation or inhibition under different experimental conditions. Two proteomic datasets were analyzed: (1) the MAFF siRNA KD (KD) dataset and (2) the MAFF mRNA OE dataset. In the MAFF OE dataset, comparisons included MAFF mRNA *versus* control mRNA under light-stress and dim-light conditions, as well as light *versus* dim-light comparisons within the control and MAFF-overexpressing groups. The same comparison strategy was applied to the MAFF KD dataset. The corresponding control under light *versus* dim-light comparison was presented in Supplementary Figure S1. For exploratory pathway analysis, candidate proteins meeting |log_2_ fold-change| > 1 and nominal *p* < 0.05 were imported into IPA. Furthermore, the selected proteins (light-responsive MAFF-associated proteins) were also subjected to IPA for canonical pathway analysis. During core analysis, the sample type filter was restricted to retina and all cell lines. Enriched canonical pathways (*p*-values generated by IPA < 0.05) and their predicted activation states were then identified. The top 10 canonical pathways ranked by significance were selected for visualization.

### 2.12. Kyoto Encyclopedia of Genes and Genomes (KEGG) and Reactome Pathway Analysis

Proteins identified as light-responsive and MAFF-associated (Figure 4A) were selected by comparing proteins differentially expressed under light stress conditions in the MAFF OE and MAFF KD arms with the corresponding light-*versus*-dim light control comparison (Figure 4A). Proteins uniquely identified in the MAFF OE arm (11 up- and 48 down-regulated proteins) and the MAFF KD arm (31 up- and 26 down-regulated proteins) were defined as light-responsive and MAFF-associated proteins. These proteins were subsequently subjected to pathway enrichment analysis using the Database for Annotation, Visualization, and Integrated Discovery (DAVID), incorporating KEGG and Reactome pathway annotations.

### 2.13. Statistical Analysis

Statistical analyses were performed using GraphPad Prism (version 11.0.1; GraphPad Software, San Diego, CA, USA) and R. For the proteomic dataset, paired two-tailed Student’s *t*-tests were used for donor-matched pairwise comparisons and exploratory candidate-protein selection for downstream pathway analyses. In addition, a factorial limma analysis with empirical Bayes moderation and Benjamini–Hochberg FDR correction was performed as a complementary analysis to assess MAFF, light, and MAFF × light effects while accounting for the paired donor structure, as described in Section 2.9. The complete limma results are provided in Supplementary Table S1. The limma results were not used as the filtering criteria for the downstream exploratory pathway analyses. Paired two-tailed Student’s *t*-tests were also used for WB band quantification. Two-way ANOVA was used to analyze differences in ROS levels between groups and to assess cell viability in the AlamarBlue assay. Data are presented as mean ± SD unless otherwise indicated. A *p* value < 0.05 was considered statistically significant for the functional assays.

### 2.14. Data Visualization

All graphical representations in this study were generated using RStudio (version 2025.09.2+418), GraphPad Prism (version 11.0.1), or BioRender.

## 3. Results

### 3.1. MAFF Overexpression Reduced Oxidative Stress and Improved Müller Cell Viability

**Figure 1:**
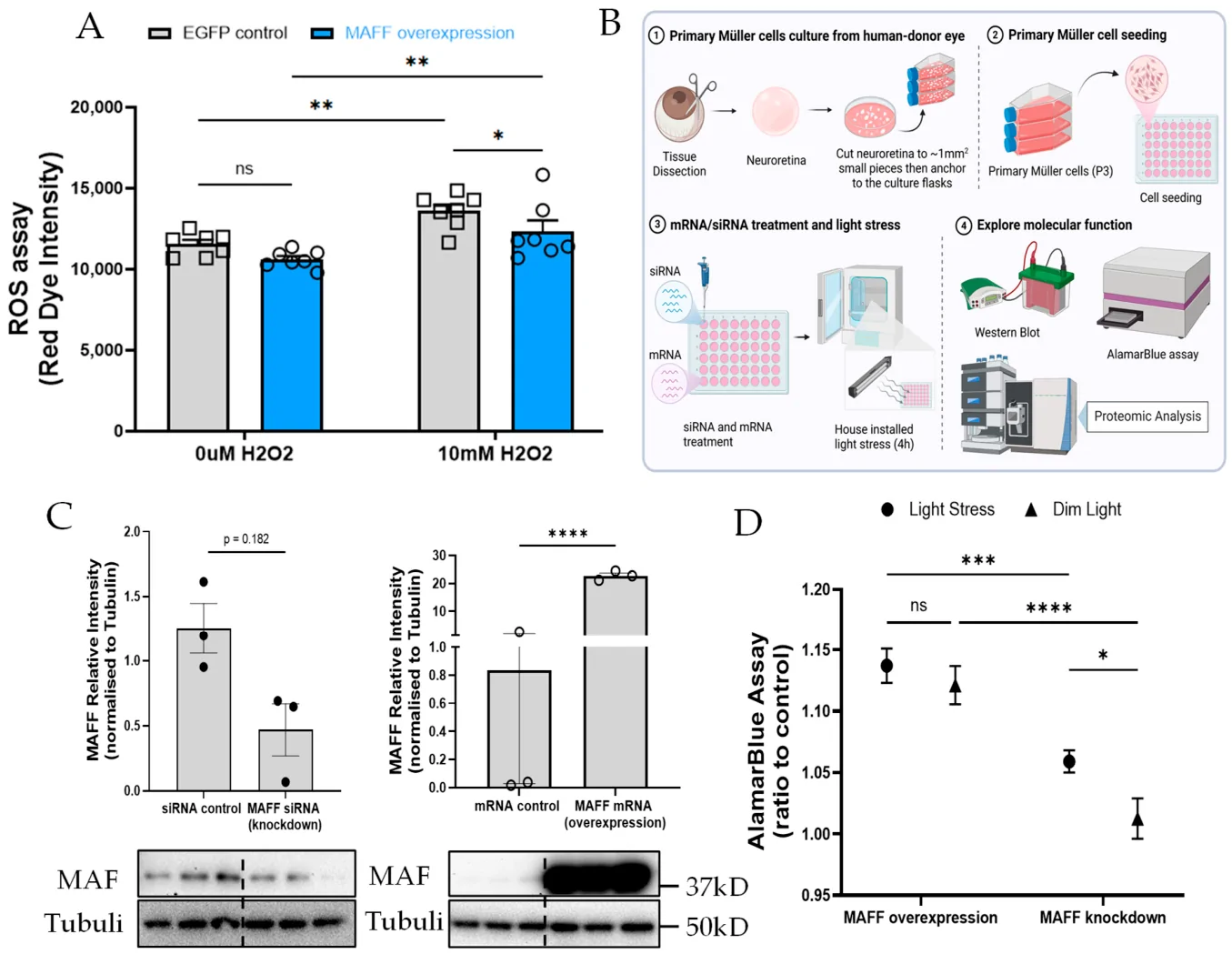
**MAFF overexpression (OE) protected Müller cells against oxidative stress (OS) and light-induced cellular injury.** (**A**) Reactive oxygen species (ROS) levels were measured under normal conditions (0 µM H_2_O_2_) and OS (10 mM H_2_O_2_) following transfection with EGFP control plasmid DNA (grey bar) and MAFF plasmid DNA (blue bar) in human primary Müller cells (huPMCs). (**B**) Experimental workflow for MAFF functional analysis in huPMCs. huPMCs were isolated from human donor eyes, cultured to passage 3 (P3), and seeded for RNA transfection. Cells were treated with MAFF siRNA for 48 h or MAFF mRNA for 24 h, together with their corresponding controls, followed by 4 h of intensive white light stress at 32,000 lux, with dim light (5 lux) used as the control condition. Functional changes were assessed by AlamarBlue assay and protein expression changes assessed by Western blot (WB). The same workflow was used for Liquid Chromatography–Tandem Mass Spectrometry (LC–MS/MS) proteomic analysis, with samples prepared separately in 6-well plates. (**C**) Validation of MAFF knockdown (KD) and OE in huPMCs by WB analysis. Relative MAFF protein expression was quantified by densitometry and normalized to α-tubulin. Representative immunoblots are shown below the corresponding quantification. (**D**) Cell viability was assessed by AlamarBlue assay in huPMCs following MAFF OE or MAFF KD under light-stress (circle) and dim-light control (triangle) conditions. Data are presented as mean ± SEM. Statistical significance was determined using two-way ANOVA with multiple-comparisons testing for (**A**,**D**); paired two-tailed Student’s *t*-test was used in comparing the protein expression in (**C**) ns, not significant; * *p* < 0.05; ** *p* < 0.01; *** *p* < 0.001; **** *p* < 0.0001.

First, we used huPMCs to explore the correlation between MAFF expression and cellular OS. HuPMCs were transfected with plasmids expressing proteins MAFF or EGFP as a negative control. Transfected cells were then treated with H_2_O_2_ to induce OS. We found no significant difference intracellular ROS levels between control and MAFF-OE groups under basal conditions. Following exposure to 10 mM H_2_O_2_, intracellular ROS levels increased by 17% in EGFP control cells compared with non-stressed controls, whereas huPMCs overexpressed with MAFF showed a 10% reduction in intracellular accumulation of ROS compared with EGFP control under OS (Figure 1A).

Based on this finding, we then established an RNA-based MAFF modulation system in huPMCs to further assess the functional role of MAFF under light stress [17]. MAFF OE was achieved by using MAFF mRNA, and MAFF KD through MAFF siRNA. Following transfection, huPMCs were maintained under dim-light control conditions or exposed to light stress (32,000 lux; white light). MAFF protein expression and cell viability were then assessed by WB and AlamarBlue assay, respectively (Figure 1B).

Changes in MAFF protein expression levels in huPMCs were assessed by WB following siRNA-mediated KD or mRNA-mediated OE. MAFF protein expression was reduced by ~62% in huPMCs treated with MAFF siRNA compared with the control siRNA. MAFF protein expression increased ~26 fold following MAFF mRNA transfection compared with mRNA control. These results validated these MAFF KD and OE models for subsequent functional and proteomic analyses (Figure 1C). The functional role of MAFF in huPMCs survival was further assessed by comparing MAFF OE and KD treated huPMC cell viability under dim-light control or light stress. MAFF OE increased relative cell viability compared with MAFF KD, with a 10% increase under dim-light control (*p* < 0.05) and a 7% increase under light stress (*p* < 0.05) (Figure 1D).

### 3.2. Proteomic Profiling Identified MAFF-Associated Changes in Müller Cells Under Light Stress

We performed global proteomic profiling using DIA LC-MS/MS following MAFF KD by siRNA (*n* = 3 different donors) and OE by mRNA (*n* = 4 different donors) in human donor-derived huPMCs to investigate the molecular changes associated with MAFF modulation. After quality control and missing value imputation, both the MAFF KD and OE datasets comprised 5907 quantified proteins. For the R-based limma and PLS-DA analyses, 5737 proteins were retained after duplicated protein entries were consolidated by averaging their log_2_-transformed abundance values. A factorial limma analysis with empirical Bayes moderation and Benjamini–Hochberg multiple-testing correction was performed, with donor, MAFF treatment, light exposure, and the MAFF × light interaction incorporated into the model; the results are provided in Supplementary Table S1. Before imputation, the overall missing value rates were 13.4% in the KD dataset and 21.6% in the OE dataset (Table A3). Comparison of MAFF protein abundance across experimental groups confirmed effective MAFF modulation. Relative to the control group, which was set as 100%, MAFF expression was reduced to 32.8% in the KD group, representing a 67.2% KD. In contrast, MAFF expression increased to 6362.4% in the OE group, corresponding to a 62.6-fold OE (Table A4).

**Figure 2:**
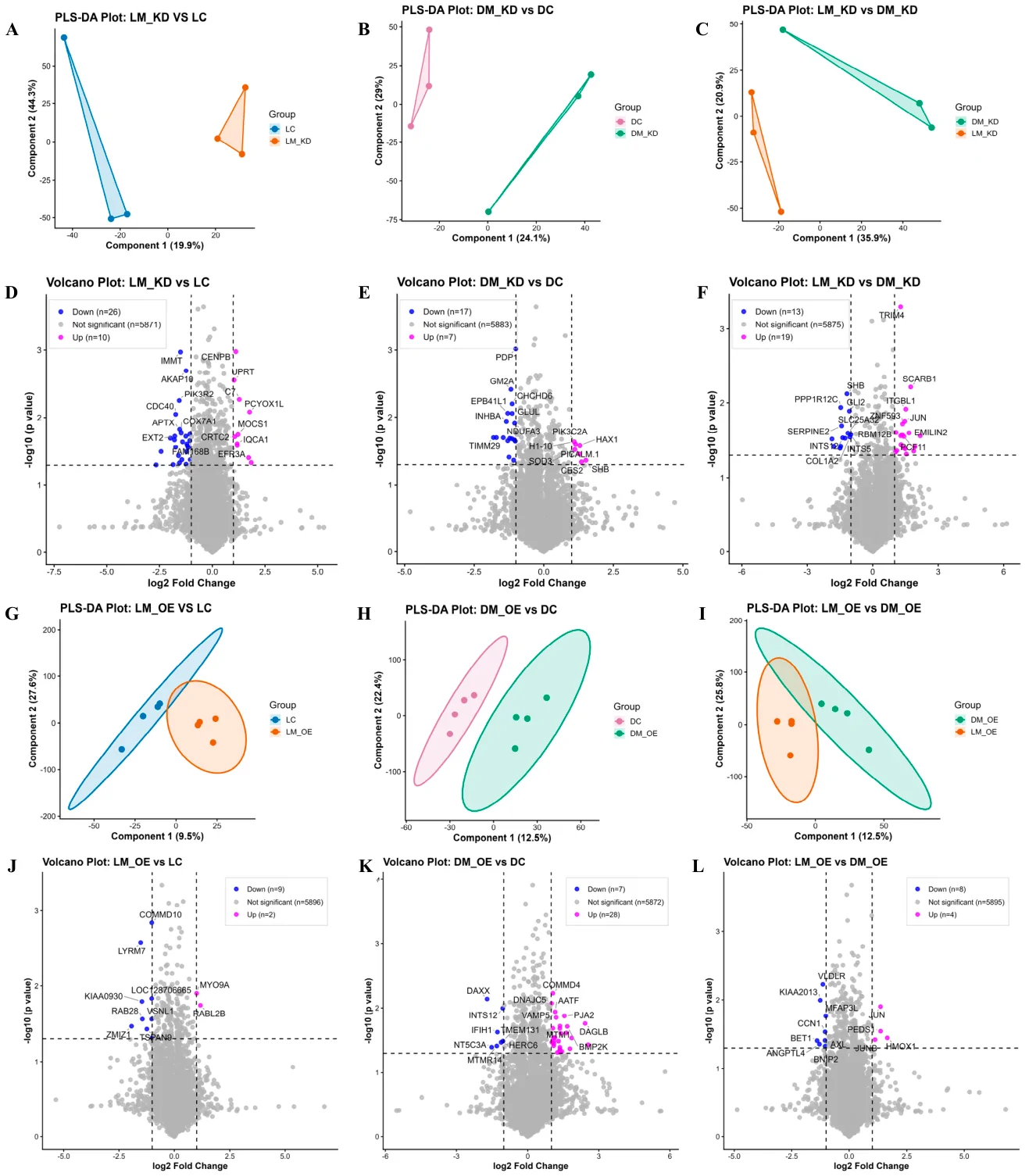
Global proteomic profiling revealed distinct protein expression patterns in Müller cells following MAFF siRNA knockdown (KD) (*n* = 3 different donors) and mRNA overexpression (OE) (*n* = 4 different donors) under light stress and dim control. (**A**–**C**) Partial least squares discriminant analysis (PLS-DA) of MAFF siRNA-treated samples: (**A**) Light-treated MAFF KD using siRNA (LM_KD) *versus* light-treated control (LC). (**B**) Dim MAFF KD treated with siRNA (DM_KD) *versus* dim control (DC). (**C**) Light *versus* dim conditions under MAFF KD (LM_KD *versus* DM_KD). Each point represents an individual donor-derived sample. Lines indicated sample grouping, and the percentage of variance explained by each component is shown on the axes. (**D**–**F**) Volcano plots of differential protein expression in MAFF siRNA-treated samples: (**D**) LM_KD *versus* LC; (**E**) DM_KD *versus* DC; and (**F**) LM_KD *versus* DM_KD. Upregulated proteins are shown in magenta, downregulated proteins in blue, and non-significant proteins in grey. Vertical dashed lines indicate fold-change thresholds (log_2_ fold-change equals −1 or 1), and the horizontal dashed lines indicate statistical significance (*p* < 0.05). Selected top differentially expressed proteins (presented in gene name) are labelled. (**G**–**I**) PLS-DA of MAFF mRNA OE samples: (**G**) Light-treated MAFF OE using mRNA (LM_OE) *versus* light-treated control (LC). (**H**) Dim MAFF OE treated with mRNA (DM_OE) *versus* dim control (DC). (**I**) Light *versus* dim conditions under MAFF OE (LM_OE *versus* DM_OE). Ellipses represent the distribution and clustering of biological replicates within each condition. (**J**–**L**) Volcano plots of differential protein expression in MAFF mRNA OE samples: (**J**) LM_OE *versus* LC; (**K**) DM_OE *versus* DC; and (**L**) LM_OE *versus* DM_OE. Color coding and statistical thresholds are as described above. In all comparisons, control groups represent condition-matched transfection controls corresponding to each treatment (i.e., siRNA control for MAFF siRNA experiments and EGFP mRNA control for MAFF mRNA OE experiments).

PLS-DA was used to visualize global patterns in protein expression across experimental groups (Figure 2A–C,G–I). Varying degrees of visual separation were observed among the comparisons. However, permutation validation did not provide statistically significant evidence of robust predictive separation (Table S2). Given the limited number of independent human donors, these PLS-DA plots are therefore interpreted only as exploratory representations of the global data structure and not as inferential evidence of group differences.

Volcano plot analysis illustrated further distinct patterns of differential protein expression across all comparisons (Figure 2D–F,J–L). We first did the comparison in the MAFF siRNA-mediated KD samples. We compared the MAFF KD and control groups under light stress (LM_KD *versus* LC; Figure 2D) to explore the molecular changes of MAFF downregulation under light stress. We identified 36 proteins meeting the exploratory selection criteria of |log_2_FC| > 1 and nominal *p* < 0.05, comprising 26 downregulated proteins (in blue), including IMMT, AKAP10, PIK3R2, CDC40, APTX, COX7A1, EXT2, and FAM168B, and 10 upregulated proteins (in magenta), including CENPB, UPRT, C7, PCYOX1L, MOCS1, IQCA1, and EFR3A.

We then compared the MAFF KD and control groups under dim-light conditions (DM_KD *versus* DC; Figure 2E) to explore the MAFF downregulation under normal conditions. Twenty-four candidate proteins were identified using the exploratory criteria in the MAFF KD group, including 17 downregulated proteins, such as PDP1, GM2A, EPB41L1, INHBA, CHCHD6, GLUL, NDUFA3, and TIMM29, and 7 upregulated proteins, including PIK3C2A, H1-10, HAX1, SOD3, PICALM, CES2, and SHB.

We then compared light stress and dim-light control in the MAFF KD group (LM_KD *versus* DM_KD; Figure 2F) to further explore the effect of MAFF downregulation on cellular stress response. We identified 32 exploratory candidate proteins, comprising 13 downregulated proteins, including SHB, PPP1R12C, GLI2, SLC25A32, SERPINE2, INTS12, INTS5, and COL1A2, and 19 upregulated proteins, including TRIM4, SCARB1, ITGBL1, ZNF593, JUN, EMILIN2, RBM12B, and PCF11. The complete list of exploratory candidate proteins with MAFF downregulation is provided in Table S5.

We also compared MAFF mRNA-mediated OE samples. MAFF OE and control group under light stress (LM_OE *versus* LC; Figure 2J) were compared to explore the molecular changes of MAFF upregulation under light stress. We identified 11 exploratory candidate proteins; comprising 9 downregulated proteins, including COMMD10, LYRM7, KIAA0930, RAB28, ZMIZ1, TSPAN9, LOC128706665, and VSNL1, and 2 upregulated proteins, including MYO9A and RABL2B.

We then compared the MAFF OE and control groups under dim-light control (DM_OE *versus* DC; Figure 2K) to explore the molecular changes by MAFF upregulation under non-stressed conditions, and identified 35 exploratory candidate proteins, including 7 downregulated proteins, DAXX, INTS12, H1-10, NT5C3A, MTMR14, DNAJC5, and TMEM131, and 28 upregulated proteins, including COMMD4, MAFF, PJA2, DAGLB, BMP2K, VAMP5, TM7M4, and TMEMC6.

To explore the cellular response to stress with MAFF upregulation further, we compared the light stress and dim-light control in the MAFF OE group (LM_OE *versus* DM_OE; Figure 2L). We identified 12 exploratory candidate proteins, comprising 8 downregulated proteins, including KIAA2013, VLDLR, MFAP3L, CCN1, BET1, ANGPTL4, and DNIP2, and 4 upregulated proteins, including JUN, PEDS1, HMOX1, and JUND. The complete list of exploratory candidate proteins with MAFF upregulation is provided in Table S5.

### 3.3. Exploratory Pathway Analysis Identifies Stress-Response Signatures Associated with MAFF Expression

**Figure 3:**
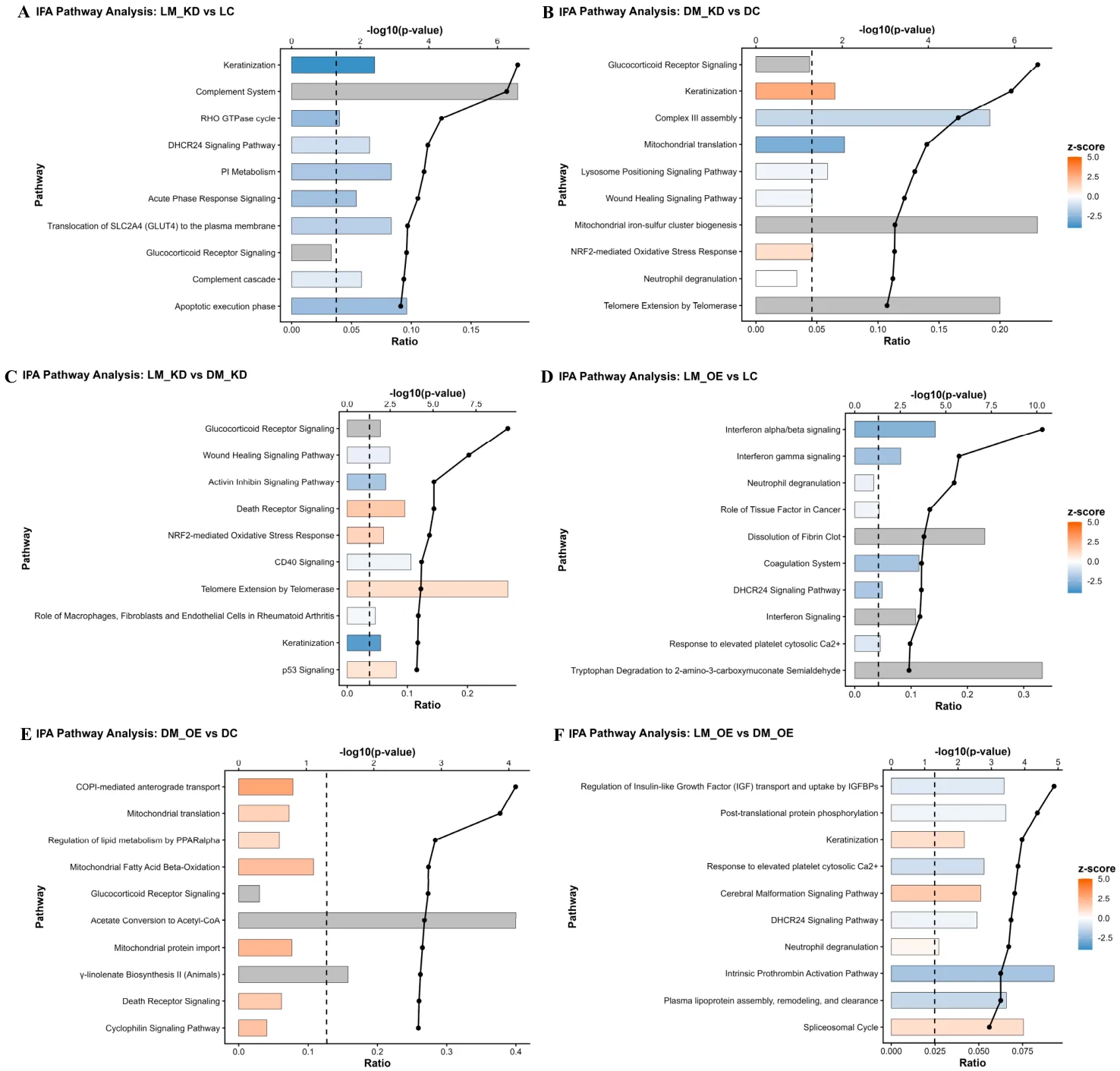
Pathway enrichment analysis (Ingenuity pathway analysis; IPA) highlighted functional changes in biological processes associated with proteomic alterations following MAFF siRNA knockdown (KD) and mRNA overexpression (OE) under light stress and dim-light control conditions. (**A**–**C**) IPA of MAFF KD (siRNA-treated) samples: (**A**) Light-treated MAFF KD using siRNA *versus* light-treated siRNA control (LM_KD *versus* LC). (**B**) Dim MAFF KD using siRNA *versus* dim siRNA control (DM_KD *versus* DC). (**C**) Light *versus* dim conditions under MAFF KD (LM_KD *versus* DM_KD). (**D**–**F**) IPA of MAFF OE (mRNA-treated) samples: (**D**) Light-treated MAFF OE using mRNA *versus* light-treated mRNA control (LM_OE *versus* LC). (**E**) Dim MAFF OE using mRNA *versus* dim mRNA control (DM_OE *versus* DC). (**F**) Light *versus* dim conditions under MAFF OE (LM_OE *versus* DM_OE). For each panel, horizontal bars represent the pathway enrichment ratio, with bar color indicating the activation z-score (orange: predicted activation; blue: predicted inhibition; white: no predicted direction; grey: z-score unavailable). The overlaid line with points represents pathway significance expressed as −log10(*p*-value). The dashed vertical line indicates the significance threshold (*p* = 0.05). Pathways are displayed in the order of significance (from top to bottom).

Pathway enrichment analysis using IPA identified multiple biological pathways associated with altered MAFF expression (Figure 3). In the comparison between the MAFF KD and control groups under light stress (LM_KD *versus* LC; Figure 3A), the most significantly IPA-selected enriched pathways included those related to cell structure and cytoskeletal organization, including Keratinization (−log(*p*) = 6.59) and the RHO GTPase cycle (−log(*p*) = 4.37); OS and lipid metabolism, including the DHCR24 signaling pathway (−log(*p*) = 3.97) and PI metabolism (−log(*p*) = 3.86); immune and inflammatory signaling, including Acute phase response signaling (−log(*p*) = 3.68) and the Complement cascade (−log(*p*) = 3.27); and cell survival and metabolic transport, including Translocation of SLC2A4 (GLUT4) to the plasma membrane (−log(*p*) = 3.38) and the Apoptotic execution phase (−log(*p*) = 3.18). All enriched pathways showed negative activation z-scores, including Keratinization (z = −3.87), the RHO GTPase cycle (z = −2.36), PI metabolism (z = −1.89), Acute phase response signaling (z = −2.12), Translocation of SLC2A4 to the plasma membrane (z = −1.63), the Complement cascade (z = −0.71) and the Apoptotic execution phase (z = −2.24).

In the comparison between the MAFF KD and control groups under dim-light conditions (DM_KD *versus* DC; Figure 3B), the significantly IPA-selected enriched pathways included those associated with mitochondrial function and energy metabolism, including Complex III assembly (−log(*p*) = 4.70) and Mitochondrial translation (−log(*p*) = 3.97); and OS and cell structure, including the NRF2-mediated oxidative stress response (−log(*p*) = 3.22) and Keratinization (−log(*p*) = 5.93). Positive activation z-scores were observed for Keratinization (z = 2.67) and the NRF2-mediated oxidative stress response (z = 1.00), whereas Complex III assembly (z = −1.34), Mitochondrial translation (z = −2.83), the Lysosome positioning signaling pathway (z = −0.33) and the Wound healing signaling pathway (z = −0.33) showed negative activation z-scores.

In the comparison between light stress and dim-light conditions in the MAFF KD group (LM_KD *versus* DM_KD; Figure 3C), the significantly IPA-selected enriched pathways included those related to cell death and stress responses, including Death receptor signaling (−log(*p*) = 5.05), p53 signaling (−log(*p*) = 4.05) and the NRF2-mediated oxidative stress response (−log(*p*) = 4.79), as well as immune and inflammatory signaling, including CD40 signaling (−log(*p*) = 4.33) and the Role of macrophages, fibroblasts and endothelial cells in rheumatoid arthritis (−log(*p*) = 4.14). Positive activation z-scores were observed for Death receptor signaling (z = 1.67), the NRF2-mediated oxidative stress response (z = 1.34), and p53 signaling (z = 0.82), whereas the CD40 signaling (z = −0.38), and the Role of macrophages, fibroblasts and endothelial cells in rheumatoid arthritis (z = −0.28) showed negative activation z-scores.

In the comparison between the MAFF OE and control groups under light stress (LM_OE *versus* LC; Figure 3D), the significantly IPA-selected enriched pathways were predominantly associated with immune and inflammatory signaling, including Interferon alpha/beta signaling (−log(*p*) = 10.30), Interferon gamma signaling (−log(*p*) = 5.73) and the Coagulation system (−log(*p*) = 3.67), together with OS signaling, including the DHCR24 signaling pathway (−log(*p*) = 3.66). All enriched pathways showed negative activation z-scores, including Interferon alpha/beta signaling (z = −2.71), Interferon gamma signaling (z = −2.12), the DHCR24 signaling pathway (z = −1.89).

In the comparison between the MAFF OE and control groups under dim-light conditions (DM_OE *versus* DC; Figure 3E), the significantly IPA enriched pathways were primarily associated with mitochondrial function and energy metabolism, including Mitochondrial translation (−log(*p*) = 3.87), Mitochondrial fatty acid β-oxidation (−log(*p*) = 2.81) and Mitochondrial protein import (−log(*p*) = 2.72); and cell death signaling, including Death receptor signaling (−log(*p*) = 2.67) and Cyclophilin signaling pathway (−log(*p*) = 2.66). All enriched pathways showed positive activation z-scores, including Mitochondrial translation (z = 1.41), Mitochondrial fatty acid β-oxidation (z = 2.00), Mitochondrial protein import (z = 2.24), Death receptor signaling (z = 1.63) and Cyclophilin signaling pathway (z = 1.90).

In the comparison between light stress and dim-light conditions in the MAFF OE group (LM_OE *versus* DM_OE; Figure 3F), the enriched pathways included those related to immune signaling, including Neutrophil degranulation (−log(*p*) = 3.52); OS signaling, including the DHCR24 signaling pathway (−log(*p*) = 3.59); and lipid metabolism, including Plasma lipoprotein assembly, remodeling and clearance (−log(*p*) = 3.28). Positive activation z-scores were observed for Neutrophil degranulation (z = 0.28), whereas the DHCR24 signaling pathway (z = −0.38), and Plasma lipoprotein assembly, remodeling and clearance (z = −1.34) showed negative activation z-scores.

Collectively, IPA canonical pathway analysis showed distinct pathway enrichment profiles according to altered MAFF expression levels. Reduced MAFF expression was associated with predicted activation of OS-related pathways, including the NRF2-mediated oxidative stress response pathway, together with predicted inhibition of mitochondrial function-related pathways, including mitochondrial translation and Complex III assembly. Elevated MAFF expression was associated with predicted activation of mitochondrial-related pathways, including mitochondrial translation, mitochondrial fatty acid β-oxidation and mitochondrial protein import under dim-light conditions, whereas under light stress it was associated with predicted inhibition of immune- and inflammation-related pathways, including interferon alpha/beta signaling, interferon gamma signaling and the DHCR24 signaling pathway.

### 3.4. Correlation Analysis Identifies Candidate Proteins Associated with MAFF-Associated Modulation of the Light-Stress Response

**Figure 4:**
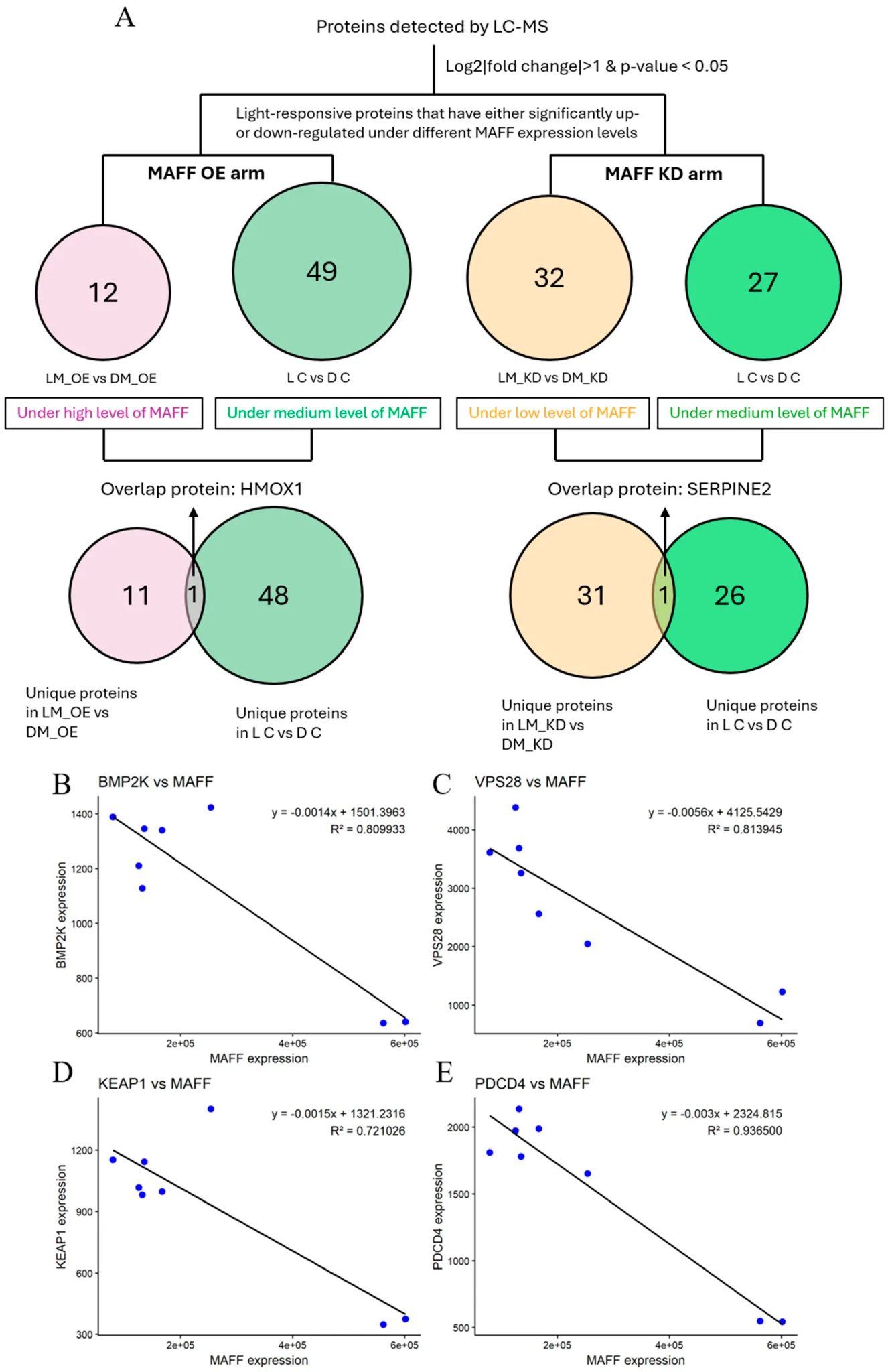
Identification of MAFF expression-associated and light-responsive proteins from proteomic data. (**A**) Proteins detected by Liquid Chromatography–Tandem Mass Spectrometry (LC-MS/MS) were first filtered based on differential expression (|log_2_ fold change| > 1) and *p*-value (*p* < 0.05) to identify light-responsive proteins under different MAFF expression conditions, including MAFF overexpression (LM_OE *versus* DM_OE), MAFF knockdown (LM_KD *versus* DM_KD), and corresponding control comparisons (LC *versus* DC). Proteins identified under high (mRNA overexpression), medium (control), and low (siRNA knockdown) MAFF expression levels were compared using Venn diagram analysis to distinguish MAFF expression–independent proteins (shared across conditions) from those specifically altered under different MAFF levels. Proteins uniquely associated with high, low, or medium MAFF expression were defined as MAFF expression-associated proteins. (**B**–**E**) Linear regression analysis of representative proteins against MAFF expression levels under MAFF overexpression comparisons. Protein expression levels for Bone Morphogenetic Protein 2 inducible Kinase (BMP2K), Vacuolar Protein Sorting-associated protein 28 Homolog (VPS28), Kelch-like ECH-Associated Protein 1 (KEAP1), and Programmed Cell Death Protein 4 (PDCD4) were nominally negative correlated with MAFF expression. Regression equations and R^2^ values are shown for each protein in panels.

To determine whether MAFF expression reshapes the proteomic response to light stress, we compared proteins that responded nominally to light exposure under different MAFF expression states rather than directly comparing protein abundance between MAFF treatment groups (Figure 4A). Exploratory candidate proteins (absolute log_2_ fold change > 1, *p* < 0.05) were first identified by comparing light-stressed and dim-light conditions within each experimental group, thereby defining the light-responsive proteome under high-, medium-, and low-MAFF expression states.

In the MAFF OE arm, 12 proteins responding to light stress under high MAFF expression (LM_OE *versus* DM_OE) were compared with 49 proteins identified in the corresponding control condition (LC *versus* DC), representing a relatively intermediate MAFF expression state. Proteins identified in both comparisons were considered core light-responsive proteins whose regulation was independent of MAFF abundance, whereas proteins identified exclusively under high MAFF expression or intermediate MAFF expression states were interpreted as MAFF-associated light-responsive proteins, whose response to light emerged specifically under elevated MAFF expression. Using this strategy, a total of 59 proteins (11 plus 48) were uniquely identified under increased MAFF expression responsive to light stress, whereas one shared protein (HMOX1) was excluded as a MAFF-independent light-responsive protein.

The same analytical strategy was applied to the MAFF KD arm. A total of 32 light-responsive proteins identified under low MAFF expression (LM_KD *versus* DM_KD) were compared with 27 proteins detected in the corresponding control condition (LC *versus* DC), representing a relatively intermediate MAFF expression state. Fifty-seven proteins (31 plus 26) responded uniquely to light under reduced MAFF expression, where as one shared protein (SERPINE2) was considered a MAFF-independent light-responsive protein and excluded from subsequent analyses.

Collectively, this comparative strategy distinguished proteins that responded to light irrespective of MAFF abundance from those whose light responsiveness varied according to MAFF expression states. The latter proteins were therefore considered candidate MAFF-associated modulators of the cellular response to light stress and were retained for downstream functional analyses.

Following this filtering strategy, 59 candidate proteins were obtained in the MAFF OE arm and 57 candidate proteins in the MAFF KD arm. Functional annotation revealed distinct biological characteristics between the two groups. Proteins associated with reduced MAFF expression were predominantly involved in extracellular matrix organization, stress adaptation and antioxidant responses, including COL1A1, COL1A2, ITGBL1, EMILIN2, MEGF6 and SOD3 (Figure S2A; Table S4). In contrast, proteins associated with elevated MAFF expression were mainly related to intracellular trafficking and membrane transport, including COG2, TRAPPC9, TRAPPC10, STX6, VPS28, DENND5A, BET1 and ATP6V0A2 (Figure S2B; Table S4). Several proteins with recognized roles in OS signaling and cell survival, including KEAP1, PDCD4, DAXX, JUN and JUNB, were also identified within the elevated MAFF-associated protein set.

To prioritize proteins most closely associated with MAFF expression further, we examined the relationship between MAFF abundance and protein expression across the medium- and high-MAFF expression states. Among the proteins analyzed, BMP2K, VPS28, KEAP1 and PDCD4 each showed inverse associations with MAFF abundance under MAFF-OE conditions (Figure 4B–E), with R^2^ values of 0.809, 0.814, 0.721 and 0.936, respectively. PDCD4 exhibited the strongest correlation among these candidates, suggesting that it may represent one of the downstream proteins most closely associated with MAFF-mediated regulation during the cellular response to OS.

## 4. Discussion and Limitations

In the present study, we first investigated the functional consequences of altering MAFF expression in huPMCs exposed to light-induced stress. We found that high expression of MAFF significantly reduced intracellular ROS accumulation and improved Müller cell survival, whereas low expression of MAFF reduced cellular viability under light-induced stress (Figure 1). OS is a major pathogenic mechanism underlying macular disease, particularly AMD, where excessive ROS disrupt retinal homeostasis and promote progressive retinal degeneration [23,24,25]. Light-induced stress induces cellular or tissue oxidative or mitochondrial stress [26]. These findings support a protective role of MAFF in Müller cells and suggest that MAFF may contribute to the maintenance of cellular homeostasis during oxidative challenge. Rather than acting solely as an antioxidant-associated transcription factor, MAFF appears to support a broader stress-resilient state in Müller cells, enabling them to maintain viability when exposed to oxidative injury.

Pathway analysis identified the NRF2-mediated oxidative stress response as one of the earliest pathways affected by MAFF deficiency. The predicted activation of this pathway following MAFF KD under both dim-light and light-stress conditions appears paradoxical, as MAFF is a well-established heterodimerization partner of NRF2 that facilitates antioxidant gene transcription. This apparent contradiction can be reconciled by considering the complexity of the NRF2 regulatory network. Activation of the NRF2-mediated oxidative stress response was also observed in the control light-stress comparison (LC *versus* DC; Figure S1A), indicating that light exposure alone is sufficient to engage this pathway under physiological MAFF expression. This observation suggests that MAFF is not the primary initiator of NRF2 signaling but instead functions within a broader OS response network. Indeed, NRF2 activation is primarily triggered by OS through KEAP1-dependent sensing [27] and can also be regulated through multiple signaling pathways, including p62/SQSTM1-mediated autophagy, PERK, PI3K/AKT and MAPK signaling [28,29]. Accordingly, the IPA canonical pathway represents coordinated changes in downstream OS-associated proteins rather than direct measurement of MAFF-dependent NRF2 transcriptional activity. Consistent with this interpretation, proteins associated with OS signaling, including SOD3, JUN, MAP2K7, GSTT2, GSTZ1, and DNAJ family members contributed to the predicted pathway activation in our dataset. Therefore, enrichment of the NRF2-mediated oxidative stress response is best interpreted as evidence of activation of an OS program rather than enhanced antioxidant function. This interpretation is further supported by the reduced viability of MAFF-deficient Müller cells despite predicted pathway activation, indicating that endogenous stress-response mechanisms were activated but were insufficient to restore redox homeostasis. Collectively, these findings suggest that loss of MAFF shifts Müller cells from a stress-resilient state into a compensatory stress-response state, characterized by activation of OS programs that nevertheless fail to prevent cellular injury.

Consistent with this loss of redox homeostasis, altered MAFF expression was also associated with widespread changes in mitochondrial-associated pathways. Low MAFF expression was associated with reduced mitochondrial-associated functions, whereas high MAFF expression was associated with restoration of mitochondrial-related functions under dim-light control. MAFF KD was accompanied by inhibition of mitochondrial-associated pathways, including mitochondrial translation and Complex III assembly, whereas MAFF OE enriched pathways involved in mitochondrial translation, mitochondrial protein import, Complex III assembly and mitochondrial fatty acid β-oxidation (Figure 3B,E). The opposing enrichment patterns observed between MAFF KD and OE suggest that MAFF expression is closely associated with mitochondrial homeostasis in Müller cells. Mitochondria are highly susceptible to oxidative damage and play a central role in cellular adaptation to OS. Impairment of mitochondrial translation can disrupt the synthesis of electron transport chain components, whereas defects in Complex III assembly may compromise oxidative phosphorylation and increase OS [30,31]. Mitochondrial dysfunction is widely recognized as a feature of retinal diseases, including AMD, where impaired energy metabolism and increased OS contribute to progressive retinal degeneration [32,33]. Together with our previous observation that MAFF modulation preserves mitochondrial membrane potential in Müller cells [17], these findings suggest that maintenance of mitochondrial homeostasis represents an important mechanism by which MAFF enhances Müller cell resilience to OS.

Disruption of redox and mitochondrial homeostasis was further accompanied by altered immune and inflammatory signaling. Several immune- and inflammation-related pathways, including interferon alpha/beta signaling and interferon gamma signaling, were predicted to be inhibited under MAFF OE during light stress (Figure 2D). Chronic OS and inflammation are closely interconnected during retinal degeneration, with activated Müller cells releasing cytokines, chemokines and ROS that further amplify retinal injury and disease progression [34,35,36]. The suppression of these pathways under MAFF OE therefore suggests that MAFF may limit secondary inflammatory responses accompanying OS.

**Scheme 1:**
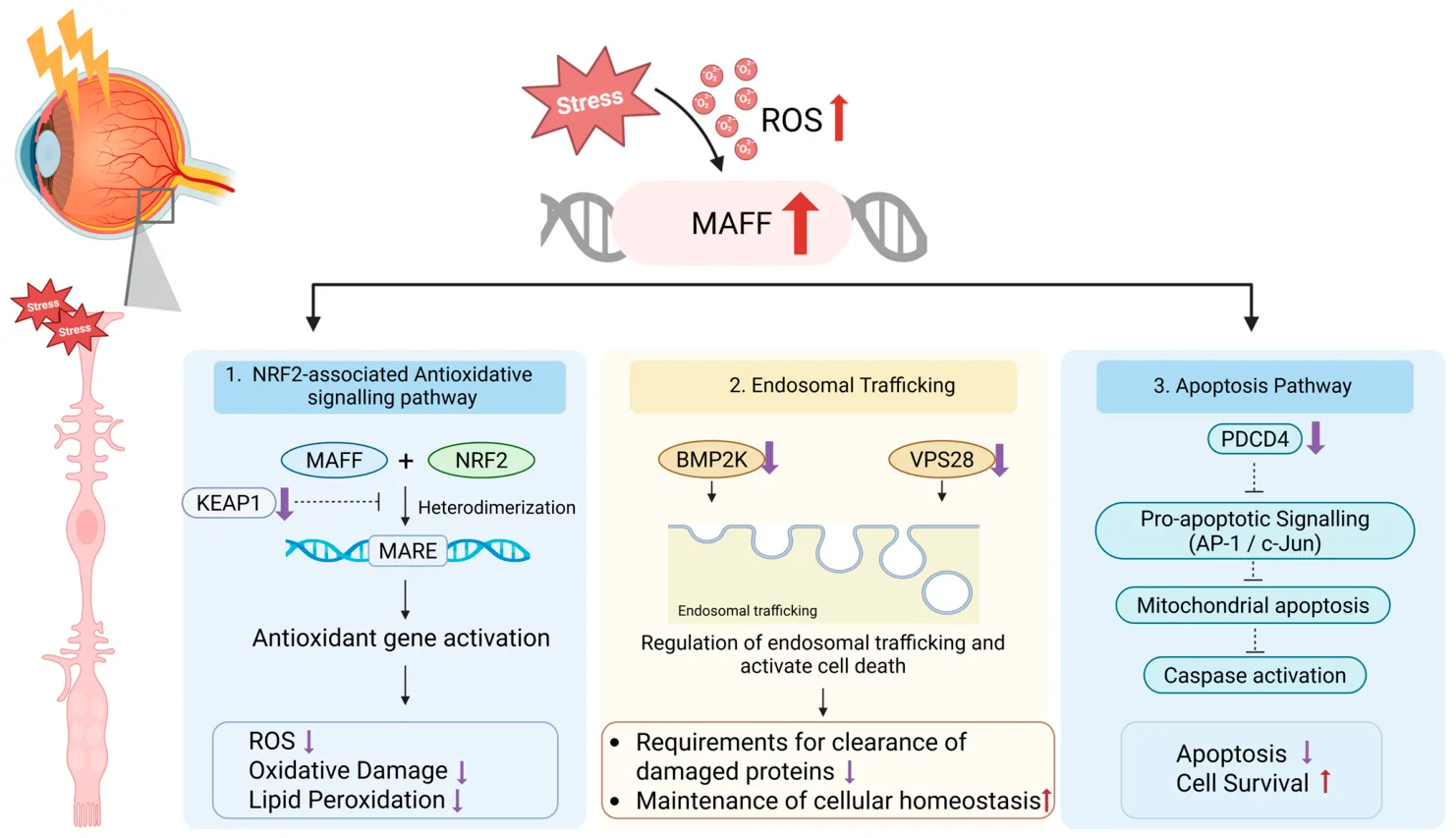
**Proposed MAFF-associated stress adaptation network in Müller cells under oxidative stress.** Correlation analysis identified Kelch-like ECH-Associated Protein 1 (KEAP1), Bone Morphogenetic Protein 2 inducible Kinase (BMP2K), Vacuolar Protein Sorting-associated protein 28 Homolog (VPS28), and Programmed Cell Death Protein 4 (PDCD4) as proteins negatively associated with MAFF expression, suggesting three major mechanisms through which MAFF may promote cellular protection. First, increased MAFF expression is associated with reduced KEAP1 levels, facilitating MAFF–NRF2 heterodimerization and activation of antioxidant response element (MARE)-dependent transcription, leading to enhanced antioxidant gene expression and reduced reactive oxygen species (ROS), oxidative damage, and lipid peroxidation. Second, increased MAFF expression is associated with decreased BMP2K and VPS28, suggesting reduced demand for OS-induced endosomal trafficking and vesicle transport responses. This may reflect improved cellular homeostasis under MAFF overexpression, with less requirement for compensatory intracellular quality-control pathways involved in the handling of damaged proteins and cellular components. Third, MAFF expression is associated with reduced PDCD4, a pro-apoptotic regulator, potentially suppressing AP-1/c-JUN-mediated apoptotic signaling, mitochondrial apoptosis, and downstream caspase activation. Together, these findings suggest that MAFF protects Müller cells against stress by coordinating antioxidant defense, vesicle trafficking and autophagy, and inhibition of apoptosis.

We examined representative proteins showing associations with MAFF expression to identify candidate molecular regulators that may underlie MAFF-associated stress resilience. Kelch-like ECH-associated protein 1 (KEAP1), Bone Morphogenetic Protein 2-Inducible Kinase (BMP2K), Vacuolar protein sorting-associated protein 28 homolog (VPS28) and Programmed cell death protein 4 (PDCD4) were all nominally negatively correlated with MAFF expression (Figure 4B–E), suggesting that increasing MAFF expression is accompanied by coordinated suppression of proteins involved in OS regulation, intracellular trafficking demand and apoptosis (Scheme 1). KEAP1 is particularly relevant because it functions as a key regulator of NRF2 stability and antioxidant signaling. KEAP1 promotes NRF2 degradation without stress, whereas OS disrupts this interaction and permits activation of antioxidant gene transcription [37,38,39,40,41]. BMP2K is a serine/threonine kinase involved in endocytic trafficking and cellular adaptation processes through Clathrin pathways [20,42,43], whereas VPS28 is a component of the ESCRT-I complex that regulates endosomal sorting, membrane protein turnover, and intracellular trafficking [44]. Increasing evidence suggests that cellular stress can remodel endocytic and endosomal trafficking pathways as part of an adaptive response aimed at maintaining homeostasis and promoting survival [45]. OS increases the burden of damaged proteins and cellular components that require processing through endosomal–lysosomal systems, thereby increasing the demand for intracellular trafficking and protein turnover mechanisms [46,47]. PDCD4 exhibited the strongest correlation with MAFF expression among the proteins examined. PDCD4 is a stress-responsive protein involved in translational regulation and apoptosis-related signaling and has been implicated in OS-induced cellular injury in several biological systems [48,49].

An important conceptual advance of the present study emerges when these findings are integrated with our previous study [17]. We previously demonstrated that, although OS induced MAFF expression in both macular and peripheral Müller cells, the magnitude of this response was significantly lower in the macula. The present study provides a potential biological framework for interpreting this regional difference, suggesting that reduced MAFF expression is associated with alterations in processes related to redox balance, mitochondrial function, inflammatory signaling, and cellular survival during OS. Together, these findings suggest that the selective vulnerability of the macula cannot be explained solely by its greater metabolic demand and cumulative light exposure, but may also reflect an intrinsically reduced capacity to maintain stress resilience. We therefore propose a model in which the lower MAFF responsiveness of macular Müller cells increases the susceptibility of the macula to OS-driven degeneration. Future studies will determine whether regional modulation of MAFF constitutes a fundamental mechanism underlying the selective vulnerability of the human macula.

Several limitations should be acknowledged. First, mechanistic interpretations were derived primarily from proteomic profiling and bioinformatic pathway analysis, which identify biological associations but do not establish direct causal relationships. Second, candidate proteins identified in the proteomic analysis were not independently validated using targeted molecular approaches such as Western blotting or immunostaining. Third, the correlation analysis was based on experimental groups representing relatively high, medium, and low MAFF expression states rather than a continuous MAFF dose–response model. Consequently, the identified protein associations should be considered exploratory and hypothesis-generating. Fourth, the proteomic analysis was based on a limited number of biologically independent human donors, and the inherent inter-donor variability of primary human Müller cells reduced statistical power for proteome-wide analysis. Given the exploratory nature of the study, nominal *p* values together with predefined fold-change criteria were therefore used to prioritize candidate proteins for downstream biological and pathway analyses, rather than to establish definitive protein-level statistical significance. These candidate proteins and associated pathways require further validation in larger independent human-donor cohorts.

## 5. Conclusions

In conclusion, our functional data support a role for MAFF in modulating Müller cell responses to oxidative and light-induced stress, with increased MAFF expression associated with reduced ROS accumulation and improved cell viability. Exploratory proteomic profiling further identified candidate molecular pathways associated with MAFF expression, including oxidative-stress responses, mitochondrial function and inflammatory signaling. Given the limited number of biologically independent human donors, these proteomic associations should be considered hypothesis-generating and require validation in larger independent donor cohorts. Together with our previous observation that MAFF expression is naturally lower in the human macula [17], these findings support further investigation of MAFF as a potential contributor to macular stress susceptibility and as a candidate for future therapeutic studies.

## Figures and Tables

**Table 1:** Primary and secondary antibodies used in WB study.

MAFF Antibody	#ab183859; Abcam, Cambridge, UK	1:1000 dilution
α/β-Tubulin Antibody	#2148S; Cell signaling, Danvers, MA, USA	1:1000 dilution
Anti-rabbit IgG, HRP-linked Antibody	#7074S; Cell signaling, Danvers, MA, USA	1:2000 dilution

## Data Availability

The original data presented in the study are openly available in iProX at PXD080464.

## Supplementary Materials

The following supporting information can be downloaded at: https://www.mdpi.com/article/10.3390/antiox15101354/s1, Figure S1: Ingenuity Pathway Analysis (IPA) of canonical pathways altered by light-induced oxidative stress (LC *versus* DC) in the MAFF overexpression and MAFF knockdown proteomic datasets; Figure S2: Functional annotation of light-responsive proteins associated with MAFF knockdown and overexpression; Table S1: Factorial limma analysis of the MAFF proteomic dataset; Table S2: Cross-validation and permutation validation of PLS-DA models across the comparisons; Table S3: Proteins meeting the exploratory differential-expression criteria shown in the volcano plot for the MAFF knockdown proteomic dataset; Table S4: Proteins meeting the exploratory differential-expression criteria shown in the volcano plot for the MAFF overexpression proteomic dataset; Table S5: Functional annotation from three different pathway analysis including IPA, Reactome and KEGG.

## Abbreviations

The following abbreviations are used in this manuscript:

BZIP	basic-leucine zipper
MAFF	MAF BZIP Transcription Factor F
MAFK	MAF BZIP Transcription Factor K
MAFG	MAF BZIP Transcription Factor G
MARE	MAF Recognition Elements
CNC	Cap‘n’Collar
NRF1	NFE2 Like BZIP Transcription Factor 1
NRF2	NFE2 Like BZIP Transcription Factor 2
BACH1	BACH Transcriptional Regulator 1
CAD	coronary artery disease
HMOX1	Heme Oxygenase 1
ER	Endoplasmic Reticulum
WB	Western Blot
ROS	Reactive Oxygen Species
LC-MS/MS	Liquid Chromatography–Tandem Mass Spectrometry
DIA	data-independent acquisition
KEGG	Kyoto Encyclopedia of Genes and Genomes
KD	Knockdown
OE	Overexpression
PLS-DA	Partial least squares discriminant analysis
VIP	variable importance in projection
PERK	Eukaryotic translation initiation factor 2-alpha kinase 3
PI3K/AKT	Phosphatidylinositol 3-kinase/AKT serine/threonine kinase 1
MAPK	Mitogen-Activated Protein Kinase
SOD3	extracellular superoxide dismutase
BMP2K	bone morphogenetic protein 2 inducible kinase
IPA	Ingenuity Pathway Analysis
KEAP1	Kelch-Like ECH-Associated Protein 1
VPS28	Vacuolar Protein Sorting-associated protein 28 Homolog
COL1A1	Collagen Type I Alpha 1 Chain
COL1A2	Collagen Type I Alpha 2 Chain
ITGBL1	Integrin Subunit Beta Like 1
EMILIN2	Elastin Microfibril Interfacer 2
MEGF6	Multiple EGF Like Domains 6
COG2	Component Of Oligomeric Golgi Complex 2
TRAPPC9	Trafficking Protein Particle Complex Subunit 9
TRAPPC10	Trafficking Protein Particle Complex Subunit 10
STX6	Syntaxin 6
DENND5A	DENN Domain Containing 5A
BET1	Bet1 Golgi Vesicular Membrane Trafficking Protein
ATP6V0A2	ATPase H+ Transporting V0 Subunit A2
DAXX	Death Domain-Associated Protein
JUN	Jun Proto-Oncogene, AP-1 Transcription Factor Subunit
JUNB	JunB Proto-Oncogene, AP-1 Transcription Factor Subunit
ESCRT-I	Endosomal Sorting Complex Required for Transport I

## Appendix A

**Table A1:** Donor characteristics, including age and sex, of human donor retinas used for primary Müller cell culture.

Donor ID	Gender	Age	Experiment
D24-0334	Male	70Y	ROS detection assay
D26-0195	Male	60Y	Western Blot
D26-0158	Male	83Y	AlamarBlue cell viability assay
D25-0748	Male	66Y	Proteomic analysis of MAFF siRNA mediated knockdown
D25-1003	Female	62Y	Proteomic analysis of MAFF siRNA mediated knockdown
D25-0844	Male	66Y	Proteomic analysis of MAFF siRNA mediated knockdown
D25-0912	Male	72Y	Proteomic analysis of MAFF mRNA mediated overexpression
D25-0619	Female	85Y	Proteomic analysis of MAFF mRNA mediated overexpression
D25-0470	Female	84Y	Proteomic analysis of MAFF mRNA mediated overexpression
D25-0422	Female	60Y	Proteomic analysis of MAFF mRNA mediated overexpression

**Table A2:** Variable Importance in Projection (VIP) scores of differentially expressed proteins identified by partial least squares discriminant analysis (PLS-DA). The table lists the Variable Importance in Projection (VIP) scores for proteins contributing to group discrimination in the PLS-DA models of the MAFF overexpression (OE) and MAFF knockdown (KD) proteomic datasets. Higher VIP scores indicate variables contributing more strongly to the observed separation in the exploratory PLS-DA model. Given the limited donor number, VIP rankings were considered descriptive and were not used as independent evidence of differential protein expression.

LM_KD *Versus* LC	DM_KD *Versus* DC	LM_KD *Versus* DM_KD	LM_OE *Versus* LC	DM_OE *Versus* DC	LM_OE *Versus* DM_OE
SERPINE2	MRPL50	COA6	MAFF	RAB34	TXNDC9
MAFF	H1-10	MRPL50	MTMR10	RPL19	STC2
TTC1	SAR1B	RCF11	RANGAP1	MAFF	AARS1
UBLCP1	NAA15	DNAJB4	ZMIZ1	RABL2B	SMARCA1
TPP2	SCPEP1	RUFY2	DDX24	OIK3C2A	AKT2
PKP2	LAMTOR2	ZMIZ1	AKAP8	TACC1	SLC4A2
TBC1D22A	TNRC6B	GLI2	RNF170	TRAPPC9	GID8
ZFO36L1	PIK3C2A	COL1A1	CD82	STXBP3	EPG5
POLR1G	NRBF2	HLA-E	PIK3C2A	PPIG	BET1
COL1A1	NAPG	SLBP	RPL7	INTS12	THOC2

**Table A3:** Summary of missing value imputation in the DIA proteomic datasets. The table summarizes the characteristics of the DIA proteomic datasets prior to missing value imputation, including the number of quantified proteins, biological samples, total protein abundance measurements, missing values, and the overall missing value rate for the MAFF overexpression (mRNA) and MAFF knockdown (siRNA) datasets. Following quality control and missing value imputation, both datasets comprised 5907 quantified proteins for downstream statistical and bioinformatic analyses.

Dateset	Proteins Quantified	Samples	Total Measurements	Missing Values	Missing Rate (%)
MAFF overexpression (mRNA)	5907	16	94,512	20,374	21.6
MAFF knockdown (siRNA)	5907	12	70,884	9517	13.4

**Table A4:** Average of the MAFF protein expression in MAFF knockdown (KD), MAFF overexpression (OE) and control (both KD and OE) groups. The control group, including both KD and OE controls, was set to 100%. Relative to control, MAFF expression was reduced by 67.2% in the KD group and increased by 62.6-fold in the OE group.

	Average in Knockdown (KD)	Average in Control (Both KD and OE)	Average in Overexpression (OE)
MAFF expression	1323.48	4035.41	25,6748.19
Percentage	32.8	100	6362.4
