# Supplementary material for: MAFF Expression Reshapes Key Stress-Response Proteins During Light-Induced Injury
Source: Antioxidants (Basel). 2026 Oct 20;15(10):1354. doi: 10.3390/antiox15101354 (PMC13701646; doi:10.3390/antiox15101354)
Supplement: Supplementary file 1 [file antioxidants-15-01354-s001.zip › Supplementary Figures.pdf]

Supplementary Figures

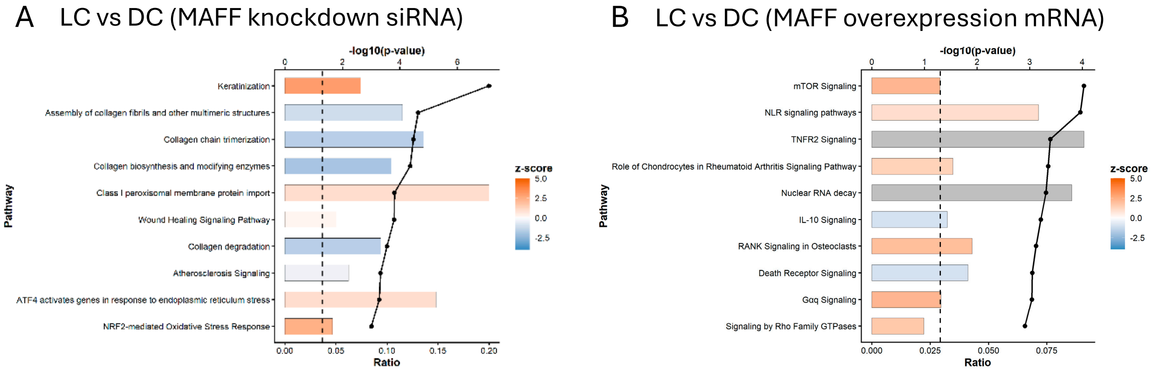

Figure S1: **Ingenuity Pathway Analysis (IPA) of canonical pathways altered by light-induced oxidative stress (LC vs DC) in the MAFF overexpression and MAFF knockdown proteomic datasets.** Canonical pathway enrichment analysis was performed by comparing light-stressed control cells (LC) with dim-light control cells (DC) in the MAFF overexpression (A) and MAFF knockdown (B) proteomic datasets. Bars represent the proportion of proteins mapped to each pathway (ratio), with colour indicating the predicted activation state (IPA z-score). The black line denotes pathway significance expressed as –log10(p-value), and the vertical dashed line indicates the significance threshold (p = 0.05).

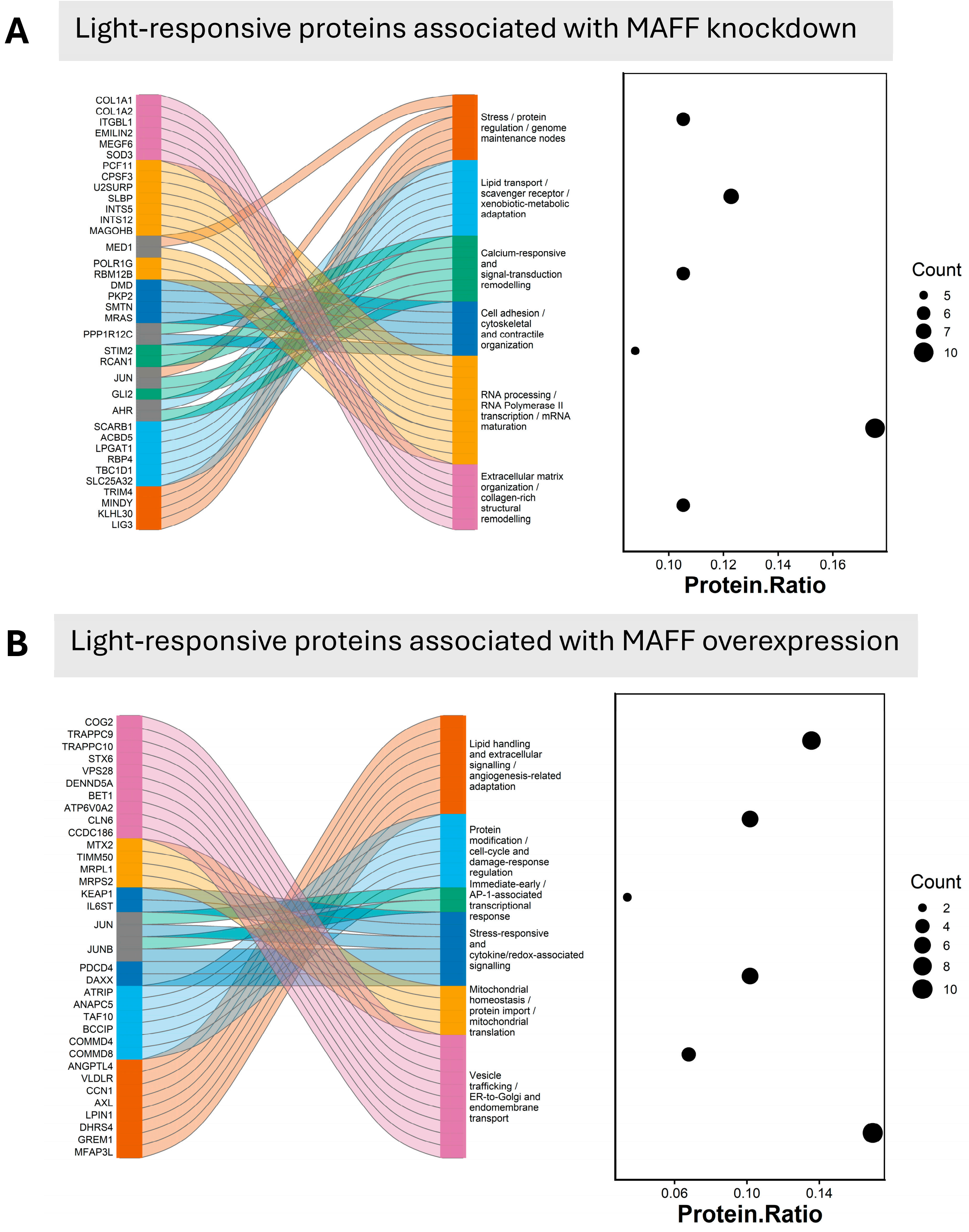

Figure S2: **Functional annotation of light-responsive proteins associated with MAFF knockdown and overexpression.** Sankey diagrams illustrate the relationships between differentially expressed light-responsive proteins and enriched functional categories following MAFF knockdown (A) or MAFF overexpression (B). Individual proteins are linked to biological functions through coloured ribbons, highlighting their contributions to distinct functional modules. Dot plots show the enrichment of each functional category, where the x-axis indicates the protein ratio and dot size represents the number of proteins assigned to the category.
